# An influential node identification method considering multi-attribute decision fusion and dependency

**DOI:** 10.1038/s41598-022-23430-3

**Published:** 2022-11-14

**Authors:** Chao-Yang Chen, Dingrong Tan, Xiangyi Meng, Jianxi Gao

**Affiliations:** 1 School of Information and Electrical Engineering, Hunan University of Science and Technology, Xiangtan, 411201 People’s Republic of China; 2 Shenzhen Institutes of Advanced Technology, Chinese Academy of Sciences, Shenzhen, 518055 People’s Republic of China; 3 Network Science Institute and Department of Physics, Northeastern University, Boston, MA 02115 USA; 4 Department of Computer Science and Network Science and Technology Center, Rensselaer Polytechnic Institute, Troy, NY 12180 USA

**Keywords:** Complex networks, Nonlinear phenomena

## Abstract

It is essential to study the robustness and centrality of interdependent networks for building reliable interdependent systems. Here, we consider a nonlinear load-capacity cascading failure model on interdependent networks, where the initial load distribution is not random, as usually assumed, but determined by the influence of each node in the interdependent network. The node influence is measured by an automated entropy-weighted multi-attribute algorithm that takes into account both different centrality measures of nodes and the interdependence of node pairs, then averaging for not only the node itself but also its nearest neighbors and next-nearest neighbors. The resilience of interdependent networks under such a more practical and accurate setting is thoroughly investigated for various network parameters, as well as how nodes from different layers are coupled and the corresponding coupling strength. The results thereby can help better monitoring interdependent systems.

## Introduction

Infrastructure networks such as power grids^1–3^, communication networks^4,5^, and transportation networks^6,7^ are usually not isolated but interdependent and coupled, forming a network of networks^8–11^. As a consequence of the dependency, random failures can easily propagate in the network, resulting in cascading effects and serious consequences. A typical example is the Italian blackout in 2003^12^, when initial failures in the grid caused other nodes in the power-grid network to fail. The resulting cascading failure left more than half of the country without power for several hours. Between 2003 and 2012, there were more than 600 power outages in the United States, affecting millions of people^13^. Such, it is of great practical significance to study the robustness of interdependent networks for building reliable interdependent systems.

Generally, the robustness of interdependent networks is mainly studied from two perspectives, namely, percolation and load capacity. In 2010, Buldyrev et al.^12^ constructed a cascading failure percolation model. They found that removing only a small proportion of initial nodes can cause failure of the entire interdependent network. Based on this model, Parshani et al.^14^ found that reducing the proportion of coupling nodes between the networks can change the percolation phase transition from being the first order to the second order, thereby improving the robustness of the network. Gao et al.^8–10^ developed an analytical framework to study the percolation of a tree-like network formed by *n* interdependent networks. They found that while for *n* = 1 the percolation transition is of second order, for *n*> 1, the network collapses as a first-order transition. Considering that the initial failure of important nodes may not be random but targeted, Huang et al.^15^ proposed a mathematical framework for understanding the robustness of interdependent networks under targeted attacks. Dong et al^16,17^. extended the framework to the scenario of targeted attacks on a general network of networks. Since then, the application of percolation theory to analyzing the robustness of interdependent networks has attracted much attention^15,18,19^.

However, the percolation model only considers the topological properties of the network, yet real-world systems often additionally carry a load (such as power or transportation). This leads to the Motter-Lai (ML) model^20^ that considers the effect of cascading failure under limited load capacity of complex networks. Attack strategy, coupling strength, load distribution strategy, network topology, etc. are the main focuses when studying load capacities. Gao et al.^21^ proposed six attack strategies for evaluating the robustness of the network. Considering the node load redistribution mechanism, Wang et al.^22^ studied the robustness of interdependent networks with different attack strategies, connection strategies, and load distribution mechanisms. While extensively studied^23–25^, the ML model assumes that the initial load and capacity follow a simple linear relation, which is unrealistic. Smaller load nodes (edges) tend to have larger capacities^26^. In light of this, Dou et al.^27^ proposed a nonlinear load capacity model for cascading failures. They found a trade-off between the cost and robustness of the interdependent networks. Chen et al.^28^ proposed a nonlinear model for cascading failure of weighted networks with overloaded edges. The model can describe the redundant capacity of edges and capture the interaction strength of nodes.

None of the above models, however, explicitly considered the fact that the distribution of load capacities of nodes are usually not totally random but are closely related to the influence of each node. For example, in a large-scale computer network, backup capacities are usually assigned to server nodes according to the importance of each server, a common approach to optimize the resources. Generally, the influence of nodes can be measured by network centrality indicators, including degree centrality^29^, betweenness centrality^30^, closeness centrality^31^, eigenvector centrality^32^, resilience centrality^33^, etc. These indicators characterize the importance of a single node in the network from different perspectives. Furthermore, a currently popular area in network science is the development of community-aware centrality measures, which identify influential nodes from the perspective of the modular structure of the network^34–36^. To improve the accuracy of identifying influential nodes, many improved centrality methods have been proposed^37–40^. The idea of multi-attribute decision-making, in particular, has been introduced for node influence evaluation. For instance, the technique for order performance by similarity to ideal solution (TOPSIS) is adopted to rank nodes based on trade-offs between existing metrics^41,42^. The analytic hierarchy process (AHP)^43^ considers several different centrality methods as multiple attributes to identify influential nodes, and so does the multi-evidence centrality (MeC)^44^ method. These methods further confirm that multi-attribute decision-making methods are more accurate than single centrality method in evaluating the influence of nodes. However, in most of these methods, multiple attributes are weighted either on an equal footing, or on a different but manually decided footing, making the methodology less objective.

This paper seeks to address the above limitations and contributes as follows. The main novelty of this work is to consider an initial load distribution that is not random but defined based on the influence of each node in the interdependent network, measured by an automated entropy-weighted multi-attribute algorithm that takes into account both different centrality measures of nodes and the interdependence of node pairs. When determining the influence of each node, the algorithm evaluates not only the node itself, but also its nearest neighbors and next-nearest neighbors. This represents another practical improvement that has seldomly been considered in the previous literature. Moreover, the load capacity of each node is determined from its initial load, not by a simple linear relation but by a general nonlinear relation. The effects of the model parameters, as well as the inter-network coupling modes and coupling strength on the robustness of the interdependent network are explored.

**Figure 1:**
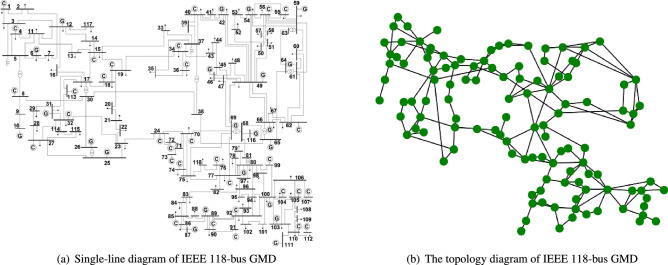
IEEE 118 power grid.

**Table 1:** Initial parameters of the interdependent network.

Parameter	ER-ER	BA-BA	ER-BA	IEEE118-BA
The number of node	500/500	500/500	500/500	118/118
System generation parameter	$p_{0}=0.008/p_{0}=0.008$	$m_{0}=2,m=2/m_{0}=2,m=2$	$p_{0}=0.008/m_{0}=2,m=2$	$-/m_{0}=2,m=2$
$<k>$	4.0/4.0	4.0/4.0	4.0/4.0	3.0/2.0

## Results

Topology is of great significance in the study of network dynamics. Typical topologies help to better analyze and control the effects of cascading failures^9,15,17^. The ER network and the BA network have different cascading failure dynamics behaviors. Therefore, this paper selects the ER network and the BA scale-free network to link, the IEEE118 actual power distribution system is abstracted into a network topology for verification. Figure 1a,b show the schematic diagram and topology of the IEEE 118 power grid, respectively. According to the different structures and characteristics of real networks, this paper generates different topologies for different situations. The network parameters are shown in Table 1. To avoid the influence of accidental factors on the experimental results, all experimental results in this paper are the average of 50 independent repeated experiments.

1. The relationship between robustness and network parameters

**Figure 2:**
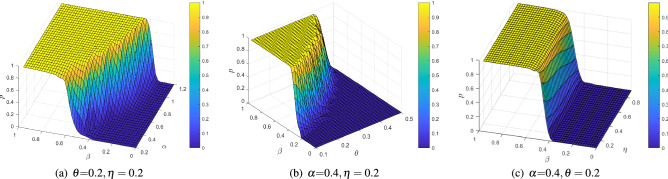
The relationship between the robustness index *p* and the network parameters $\alpha ,\beta ,\eta ,\theta$.

Consider the BA-BA network as an example. Set the number of sub-network nodes $N=500$, and the average degree $<\hbox {k}>=4$. The effect of the network parameters $\alpha ,\beta ,\eta ,\theta$ in the nonlinear model (see “Methods”) on the robustness evaluation indicator *p* is investigated. As shown in Fig. 2a, the selected parameter $\alpha = 0.4, \eta = 0.2$ remains unchanged, and *p* increases gradually with the increase of $\alpha$. The larger $\alpha$ is, the smaller the increment of $\beta$ required for *p* to jump from a small value to a large value, and the steeper the trend. That is to say, better robust performance can be obtained by only adding a smaller capacity, which can effectively resist cascading failures at a lower cost. However, when a critical phenomenon occurs, *p* no longer increases substantially with the increase of $\beta$, but gradually approaches 1. It means that the node failure at this time will not cause a large-scale spread of the failure, and the network can maintain good connectivity. Setting the parameter $\alpha = 0.4,\eta = 0.2$, it can be observed from Fig. 2b that as the load parameter $\theta$ increases, *p* grows more slowly and the network exhibits worse connectivity. This conclusion is actually in line with the characteristics of actual networks. That is, the load and capacity of a node are not simply linear, and many nodes with smaller capacity have larger redundant capacity. For example, a road network with greater unoccupied capacity in areas of light loading exhibits less efficient behavior, but this feature may provide alternative routes for congested traffic. Setting the parameter $\alpha = 0.4,\theta = 0.2$, Fig. 2c shows that the sensitivity of *p* to $\eta$ is not high, but it still offers a certain law. As $\eta$ increases, the connectivity of the entire system becomes worse. In other words, the greater the influence of dependent nodes, the less robust the network will be. Therefore, a reasonable selection of network parameters can improve the robustness of the network while appropriately reducing the cost.

**Figure 3:**
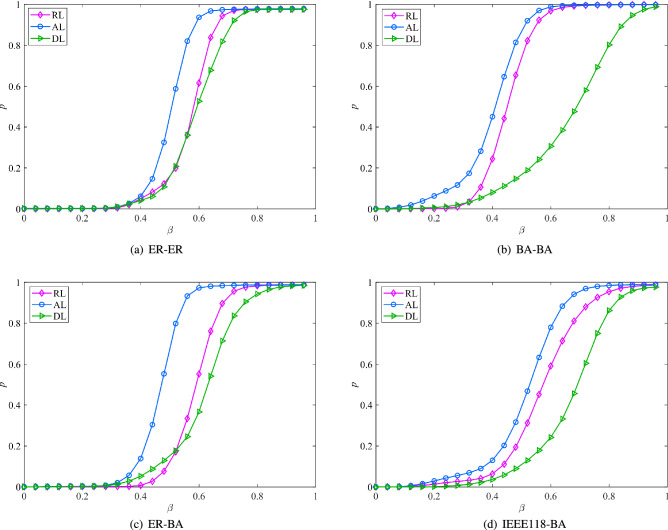
Robustness of interdependent networks under different coupling modes.

2. Robustness analysis of interdependent networks under different coupling modes

Set the initial load and capacity parameter $\theta = 0.2,\eta = 0.2,\alpha = 0.4$. The interdependent network is constructed according to three coupling modes, namely assortative link (AL), disassortative link (DL), and random link (RL)^45^. By adjusting the capacity parameter $\beta$ of nodes, the robustness rules of different interdependent networks under different coupling modes are explored. The simulation results are shown in Fig. 3. Obviously, the robustness of different interdependent networks in the three coupling modes satisfies: ${p_{AL}}> {p_{RL}} > {p_{DL}}$. Assortative link means that the nodes with larger betweenness in two subnets are connected to each other. Since nodes with larger betweenness have a larger capacity threshold, the interdependent network has a stronger ability to carry loads, and nodes are less prone to collapse. Therefore, the interdependent network is the most robust under assortative coupling. Moreover, all learning curves exhibit a three-stage characteristic with the node capacity parameter $\beta$. That is, adjusting the node capacity threshold within an appropriate interval can effectively improve the robustness of the interdependent system. Besides, the learning curves of assortative link and disassortative link in Fig. 3b–d have a large difference, but this difference is small in Fig. 3a. The reason for this phenomenon is that the degree distribution of the BA network has strong heterogeneity, while the degree distribution of the ER network is relatively uniform. This difference in network topology results in the difference in the robustness effects of the coupling modes. Therefore, when building an interdependent system, no matter what type of network the subsystems belong to, choosing the assortative coupling can maximize the robustness of the interdependent system. If it is necessary to select a disassortative mode for coupling, the coupling between subsystems with strong heterogeneity should also be avoided as much as possible.

**Figure 4:**
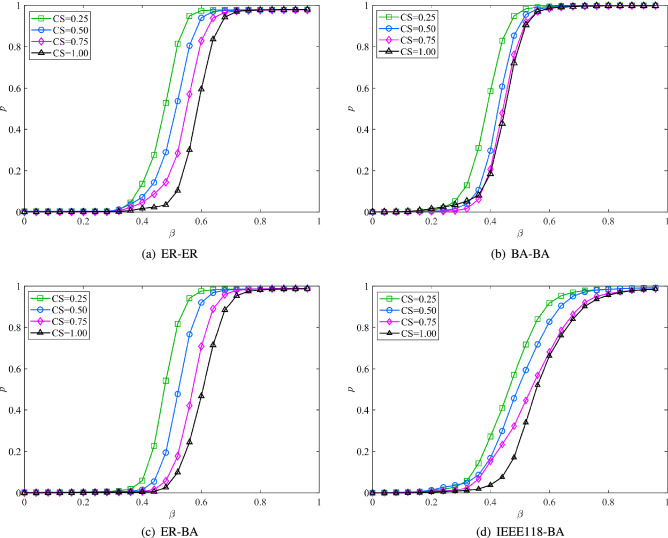
Robustness analysis of interdependent networks under different coupling strengths.

3. Robustness analysis of interdependent networks under different coupling strengths

Keep the initial parameters of the network unchanged, and choose a random link method to couple the sub-networks. Set the coupling strength parameters to 0.25, 0.5, 0.75, and 1, respectively. The relationship between the robustness of the interdependent network and the coupling strength is shown in Fig. 4. As the coupling strength increases, the robust performance gets worse. To obtain better robustness, the required capacity parameter $\beta$ is larger. That is to say, the stronger the coupling strength of the interdependent network, the easier the cascading influence will spread in the network, and the easier the whole system will collapse. This echoes the results obtained by Parshani et al^14^. In addition, while all the curves in Fig. 4 exhibit three-stage characteristics, the connectivity *p* of the curves in Fig. 4d grows slowly with the parameter $\beta$. This is because the average degree of the two subnets is quite different, and the network similarity is low. Therefore, selecting sub-networks with high similarity for assortative coupling of key nodes has practical significance for improving the reliability of interdependent systems.

4. Comparative analysis of node influence identification methods

**Figure 5:**
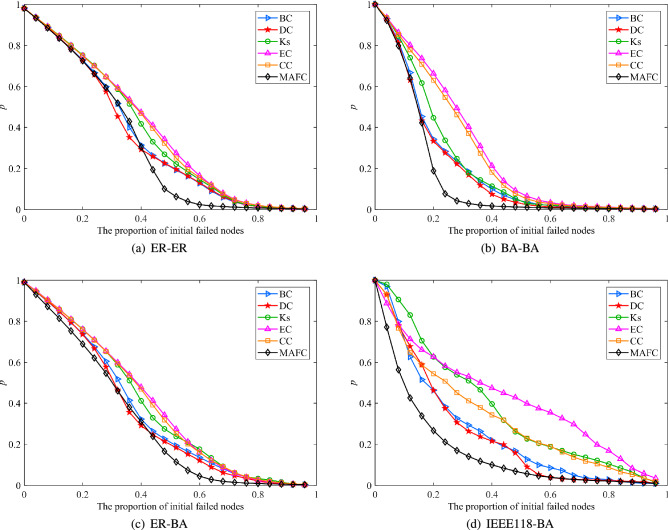
Comparative analysis of influence node identification methods.

Maintain the initial parameters and coupling methods unchanged. Selecting a proportion of initial failure nodes, the cascading failure of the interdependent network under the proposed nonlinear load capacity model is analyzed, and the proposed MAFC centrality method is compared with the classical centrality methods. As shown in Fig. 5, the trends of all learning curves are basically the same, which indicates that different centrality methods have a certain similarity in identifying influential nodes. Rank node influence according to different centrality methods. It can be observed that MAFC makes the connectivity of the network decay faster. In different types of dependent networks, the ranking accuracy of influential nodes obtained by the MAFC method is higher than that of the general centrality method. But obviously, there are some distinctions between Fig. 5a–d. The degree distribution of the network has a great impact on the ranking results. The more uniform the degree distribution of the network, the smaller the difference in ranking results. By simulating different types of interdependent networks, it means that MAFC can not only accurately identify influential nodes in interdependent networks, but also can be widely applied to different complex interdependent networks. Therefore, the proposed MAFC outperforms previous centrality methods in the context of dependent networks, which has important implications for operating and controlling complex interdependent networks.

5. Model comparison

**Figure 6:**
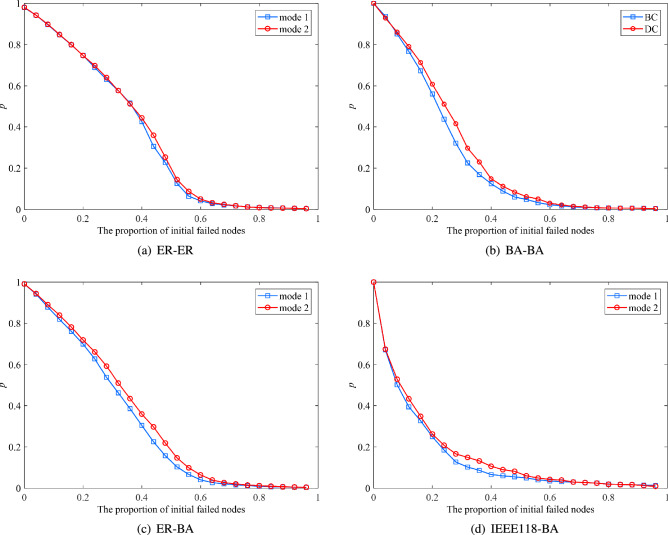
Model comparison.

Hold the initial parameters and network coupling mode unchanged. Select MAFC to sort node influence. The ML linear model (Model 1) and the proposed nonlinear model (Model 2) are compared and analyzed. As shown in Fig. 6, Model 2 has stronger connectivity than the ML linear model as the proportion of initial failed nodes increases. That is, compared with the ML linear model, the nonlinear model proposed in this paper, which considers the dependencies and actual flow, is more reliable and robust. Therefore, when modeling interdependent networks, this model is chosen to be more robust against cascading failures while being realistic.

## Discussion

Considering the traffic characteristics of the actual network and the nonlinear relationship between load and capacity, a nonlinear model of cascading failure based on node betweenness and node pair dependencies is proposed. Furthermore, the following conclusions are mainly drawn from the two aspects of the robustness and centrality of the interdependent network.

1. In the nonlinear cascade failure model proposed for interdependent networks, the choice of initial parameters makes a difference in the robustness of the network. Increasing the capacity parameter in a small range can steeply improve the network robustness, but the network robustness does not change significantly after a certain threshold is exceeded. An increase in capacity parameters means an increase in network costs. Choosing appropriate network parameters can improve network robustness and reduce network costs.
2. Employing betweenness coupling instead of degree coupling, some robust laws of interdependent networks are obtained. The robustness of the assortative link network is the highest, and the robustness of the disassortative link network is the worst. The higher the coupling strength, the worse the robustness of the interdependent network. Therefore, try to select a small number of nodes with high influence to couple with each other, while increasing the similarity between networks.
3. A multi-attribute decision fusion centrality (MAFC) algorithm is proposed based on interdependent networks. This method has higher accuracy than the single centrality method in identifying influential nodes, which provides a strong reference for the protection and backup of influential nodes in interdependent systems.
4. Since the nodes sorted by MAFC are more destructive to the network, MAFC is selected to sort the network nodes. On this basis, the proposed nonlinear model and the ML linear model are compared. This model was found to be more reliable than the ML linear model.

## Methods

### Cascading failure propagation model for interdependent networks

1. Build subnet topology

Based on complex network theory, the elements in the sub-network are regarded as nodes, and the relationship between elements is abstracted as edges, then the subsystem can be represented by a graph as

1 $$\begin{aligned} G = (V,E), \end{aligned}$$

where $V = \{ {v_i}\left| {i = 1,2, \ldots ,N} \right. \}$ is the set of all nodes in the network, and $E = \{ {e_k} = ({v_i},{v_j})\left| {k = 1,2, \ldots ,z} \right. \}$ is the set of all connected edges of the network.

2. Define the coupling relationship

The betweenness indicates the ratio of the number of paths passing through the node among all the shortest paths in the network to the total number of shortest paths. It reflects the importance of passing through the node between all pairs of nodes. The degree denotes the number of all edges connected to the node. But the degree treats all connected edges as equivalent, which is not practical. For example, in the power system, in addition to geographical factors, the betweenness of nodes can better reflect the voltage load of the transformer. The traffic in the network is transmitted according to the shortest path, which can save cost and reduce transmission loss. Therefore, choosing the node betweenness to characterize the importance of system elements will be better. The node betweenness $b_i$ can be expressed as

2 $$\begin{aligned} {b_i} = \sum \limits _{s \ne i \ne t} {\frac{{{\sigma _{st}}(i)}}{{{\sigma _{st}}}}}, \end{aligned}$$

where ${\sigma _{st}}$ is the shortest path of $s \rightarrow t$; ${\sigma _{st}}(i)$ is the shortest path of $s \rightarrow t$ through node *i*.

There are three main types of coupling relationships based on the degree of nodes, namely assortative link (AL), disassortative coupling (DL), and random link (RL). Sort all nodes in network A and network B according to the degree value. Assortative link refers to selecting nodes with high degree values in network A and network B to link in sequence. Disassortative link refers to linking nodes with high degree values in network A to nodes with low degree values in network B. Random link refers to randomly selecting one node in each of the two subnets to link. Similarly, this paper uses the betweenness of nodes as the coupling basis to construct the relationship, and the steps are as follows:

1. Calculate the node betweenness of subnet A and subnet B, respectively, expressed as ${b_{Ai}}$ and ${b_{Bi}}$;
2. Arrange the betweenness of nodes in subnet A and subnet B in descending order, namely: ${b_{A1}}> {b_{A2}}> \cdots {b_{Ai}}> \cdots > {b_{AN}}$, ${b_{B1}}> {b_{B2}}> \cdots {b_{Bj}}> \cdots > {b_{BN}}$. If there are nodes with the same betweenness, they are sorted randomly.
3. Select a certain proportion of nodes, and construct an interdependent network according to assortative link, disassortative link, and random link.

3. A cascading failure nonlinear model

In the interdependent network, the cascading failure propagation mechanism mainly includes two aspects: on the one hand, the redistribution of the load of nodes within each subnet causes adjacent nodes to fail due to overloaded loads, which in turn causes cascading failures within the subnet. On the other hand, the failure of sub-network nodes leads to the removal of coupled nodes, which in turn causes cascading failure propagation between networks. These two propagation mechanisms work together to facilitate the propagation of cascading faults in the interdependent network.

The load is the amount of traffic such as resources and information that a node carries when the network is running, and the capacity reflects the inherent ability of the node to handle the load. Based on the one-to-one interdependent network, the interaction between nodes in the sub-networks when cascading failures occur is considered. Taking subnet A as an instance, this paper redefines the initial load of a node as follows:

3 $$\begin{aligned} {L_{Ai}} = {\left( {(1 - \eta ){b_{Ai}} + \eta {b_{Bj}}} \right) ^\theta }, \end{aligned}$$

where ${L_{Ai}}$ represents the initial load of node *i* in subnet A, and ${b_{Ai}}$ and ${b_{Bj}}$ are the betweenness of the dependent node pair $i \leftrightarrow j$, respectively. $\theta$ denotes the load adjustment parameter, and $\eta$ reflects the degree of influence of node *j* in subnet B on node *i* in subnet A, $0< \eta < 1$. The larger the $\eta$ is, the more influenced by the dependent node.

The load and capacity of most practical networks, such as transportation networks and power grids, show a nonlinear relationship. Nodes with smaller capacity in the network instead have larger idle capacity^26^. Therefore, the relationship between node capacity and initial load can satisfy the following nonlinear relationship:

4 $$\begin{aligned} {C_i} = {L_i} + \beta L_i^\alpha , \end{aligned}$$

where ${C_i}$ is the capacity of node *i*, and $\alpha ,\beta$ are the capacity adjustment parameters. The model degenerates to the ML^20^ linear model when $\alpha = 1$.

Assuming that node *i* in network A fails due to attack or overload, the load of the failed node needs to be distributed to neighboring nodes. If node *j* is a neighbor node of node *i*, according to the principle of partial load redistribution^46^, the load received by node *j* from failed node *i* can be described as

5 $$\begin{aligned} \Delta {L_j} = {L_i}\frac{{{L_j}}}{{\sum \nolimits _{m \in {\Gamma _i}} {{L_m}} }}, \end{aligned}$$

where *m* denotes all neighbor nodes of node *i*, and ${\Gamma _i}$ is the set of all neighbor nodes of node *i*.

When the load of the node satisfies

6 $$\begin{aligned} {L_i} + \Delta {L_i} < {C_i}, \end{aligned}$$

the node works fine. Otherwise, the node is overloaded, and the load of the node is redistributed according to the above principles.

### Node influence identification

1. Classic centrality evaluation indicators

#### Degree centrality

Degree centrality symbolizes the ability of a node to interact with its neighbors. It describes the immediate impact of this node on a local scale. Nodes with greater centrality are generally considered to be more important. Degree centrality is defined as the ratio of the number of nodes directly connected to node *i* to the maximum possible number of nodes connected to node *i*.

7 $$\begin{aligned} DC(i) =\frac{{{k_i}}}{{N - 1}}, \end{aligned}$$

where ${k_i}$ represents the degree of node *i*, and *N* represents the number of nodes in the network.

#### Betweenness centrality

Betweenness centrality reflects the ability of nodes to control the network flow along the shortest path in the network. Betweenness centrality is expressed as

8 $$\begin{aligned} BC(i) = \frac{{2{b_i}}}{{N(N - 1)}}, \end{aligned}$$

where ${b_i}$ denotes the betweenness of node *i*, and $N(N - 1)/2$ is used to normalize the betweenness value.

#### Eigenvector centrality

The definition of eigenvector centrality considers both the quantity and quality of neighbor nodes. The way a node increases its importance is to connect many other important nodes.

9 $$\begin{aligned} EC(i) = \frac{1}{\lambda }\sum \limits _{j = 1}^N {{a_{ij}}{x_{ij}}}, \end{aligned}$$

where ${a_{ij}}$ is an element of the adjacency matrix A. $\lambda$ is the largest eigenvalue of A. ${x_j}$ represents the jth largest eigenvector sorted after the eigenvectors of A are normalized.

#### Cloneseness centrality

The closeness centrality of a node can be expressed as the inverse of the total length of the shortest path from node *i* to all other nodes in the network. The larger the centrality value, the closer the node *i* is to the center of the network, and the node *i* occupies an important position in the network.

10 $$\begin{aligned} CC(i) = \frac{{N - 1}}{{\sum \nolimits _{j = 1}^N {{d_{ij}}} }}, \end{aligned}$$

where $d_{ij}$ represents the shortest path length from node *i* to node *j*.

#### K-shell decomposition

The K-shell (Ks) decomposition method^47^ recursively removes nodes with degrees less than or equal to K, and the removed nodes simultaneously obtain a corresponding Ks value. The sub-network consisting of nodes with Ks value equal to K is called the K-shell of network G. K-shell decomposition can determine the position of a node in the network. It peels off the peripheral nodes layer by layer, and the node in the inner layer has a greater impact.

2. Influential node identification method

#### Main idea

The influence of nodes is closely related to the heterogeneity of the network. Researchers have proposed many centrality measurement methods for node influence identification, which characterize the importance of nodes from different perspectives. However, these methods are all aimed at a single network, and the network structure in the real world is complex and interdependent. It is hard to use a single metric to describe how important a node is in an interdependent network. Based on the idea of multi-attribute decision-making, multiple centrality indexes are adopted for a comprehensive evaluation, which makes up for the one-sidedness of a single index evaluation. Different attributes often have different effects on nodes. In order to avoid the subjectivity of artificially assigning weights to centrality indicators, the entropy weight method solves the problem of weight distribution of different centrality indicators well. From the perspective of information dissemination, information entropy can represent the value of information. Generally, when a message has a high probability of occurrence, it means that it is widely spread. The entropy weight method can measure the size of the data difference within the index. The greater the difference, the greater the information content of the indicator. The combined centrality is obtained by fusing the weight assigned to each attribute with the normalized centrality value. To overcome the deficiency of the interaction between adjacent nodes, a new node influence is obtained based on the principle of nearest and next nearest neighbors. In the interdependent network, the influence of a node is affected not only by the sub-network structure where the node is located but also by its coupled nodes. Therefore, it is essential to construct a linear function to map the influence of nodes to the other side of the subnet.

As shown in Table 2, three important features that affect the influence of nodes are summarized from five classic importance identification methods, namely locality, globality and node location. In this paper, the above five centrality indexes are adopted to compute the centrality of multi-attribute decision-making.

**Table 2:** Influential node identification methods for different node attributes.

Category	Locality	Globality	Location
Centrality methods	DC, EC	BC, CC	Ks

#### Computational process

Suppose a sub-network with *N* nodes, consider each node in the sub-network as a scheme and regard multiple centrality indicators for evaluating the influence of nodes as attributes of the scheme. Furthermore, the problem of node influence evaluation is transformed into a multi-attribute decision-making problem. The set of all decision-making schemes can be represented as $U = \{ {u_1},{u_2}, \ldots ,{u_N}\}$. If there are *M* centrality indicators for evaluating the influence of each node, the corresponding scheme attribute set is denoted as $F = \{ {f_1},{f_2}, \ldots ,{f_M}\}$.

1. Establish a multi-attribute decision matrix, which consists of N nodes and M centrality indicators, denoted as $X = {({x_{ij}})_{N \times M}}$.
  11 $$\begin{aligned} X = \left[ {\begin{array}{cccc} {{x_{11}}}&{}{{x_{12}}}&{} \cdots &{}{{x_{1M}}}\\ {{x_{21}}}&{}{{x_{21}}}&{} \cdots &{}{{x_{2M}}}\\ \vdots &{} \vdots &{} \ddots &{} \vdots \\ {{x_{N1}}}&{}{{x_{N2}}}&{} \cdots &{}{{x_{NM}}} \end{array}} \right] , \end{aligned}$$
  where ${x_{ij}}(i = 1,2, \ldots ,N;j = 1,2, \ldots ,M)$ in the decision matrix *X* represents the jth attribute of the *i*th node.
2. Normalize the decision matrix. The value of each evaluation index varies greatly due to different dimensions. Before evaluating the influence of nodes, it is necessary to eliminate the different dimensions of the index. The normalized matrix is denoted as $Y = {({y_{ij}})_{N \times m}}$.
  12 $$\begin{aligned} {y_{ij}} = {{{x_{ij}}} \Bigg / {\sqrt{\sum \limits _{l = 1}^N {{{({x_{lj}})}^2}} } }}, \end{aligned}$$
3. Compute the information entropy of each centrality index. Suppose $p = ({p_1},{p_2}, \ldots ,{p_n})$ is a probability vector, $1 \le {p_i} \le 1$ and $\sum \limits _{i = 1}^n {{p_i} = 1}$. Information entropy^48^ is defined as
  13 $$\begin{aligned} I(p) = - \sum \limits _{i = 1}^n {{p_i}} \log ({p_i}). \end{aligned}$$
  On this basis, the information entropy of each centrality index can be calculated as follows:
  14 $$\begin{aligned} {R_j} = - K\sum \limits _{i = 1}^N {{P_{ij}}} \ln {P_{ij}}, \end{aligned}$$
  where $$K = {1 / {\ln N}}$$, $${P_{ij}} = {y_{ij}}/\sum \nolimits _{j = 1}^M {{y_{ij}}}$$; if $${P_{ij}} = 1$$, $${P_{ij}} = (1 + {y_{ij}})/(\sum \nolimits _{j = 1}^M {(1 + {y_{ij}})} )$$.
4. Compute the entropy weight of each indicator. Generally, the smaller the information entropy value is, the more information the index provides, and the larger the weight is. The weight assigned to each attribute is
  15 $$\begin{aligned} {\omega _j} = \frac{{1 - {R_j}}}{{\sum \nolimits _{j = 1}^M {(1 - {R_j})} }}. \end{aligned}$$
5. Compute the combined centrality of nodes. The entropy weight assigned by each centrality is fused with the normalized centrality value, and the combined centrality of each node is obtained as
  16 $$\begin{aligned} {H_i} = \sum \limits _{j = 1}^M {{\omega _j}} {y_{ij}}. \end{aligned}$$
6. Determine the influence of a node. Based on the principle of nearest and next nearest neighbors^49^, the degree is substituted by the combined centrality of nodes:
  17 $$\begin{aligned} Q(u) = \sum \limits _{w \in \Gamma (u)} {N(w)} \end{aligned},$$
  18 $$\begin{aligned} {C_L}(v) = \sum \limits _{u \in \Gamma (v)} {Q(u)}, \end{aligned}$$
  where ${C_L}(v)$ is the combined centrality of node *v*, $\Gamma (u)$ is the set of first-order neighbor nodes of node *u*, and *N*(*w*) is the number of first-order and second-order neighbor nodes of node *w*. The resulting one-sided subnet influences are denoted by ${\varphi _A}({v_i}),{\varphi _B}({v_j})$, respectively.
7. Construct a linear mapping function. In an interdependent system, the influence of a node is related to the network topology where it is located and the topology of the coupled network. Taking the power system as an example, assume that *A* and *B* are the power grid and the communication grid, respectively. Usually, the power node and the communication node are not completely one-to-one coupled, and the coupling relationship matrix is represented as:
  19 $$\begin{aligned} {W_{A - B}} = \left[ {\begin{array}{cccc} {{w_{11}}}&{}{{w_{12}}}&{} \cdots &{}{{w_{1Q}}}\\ {{w_{21}}}&{}{{w_{22}}}&{} \cdots &{}{{w_{2Q}}}\\ \vdots &{} \vdots &{} \ddots &{} \vdots \\ {{w_{P1}}}&{}{{w_{P2}}}&{} \cdots &{}{{w_{PQ}}} \end{array}} \right] , \end{aligned}$$
  where *P* and *Q* are the number of nodes in the power grid and communication network, respectively. $w_{ij}$ represents the connection relationship between the nodes of the power grid and the communication network. If node *i* is connected to node *j*, ${w_{ij}} = 1$; otherwise ${w_{ij}} = 0$.

**Figure 7:**
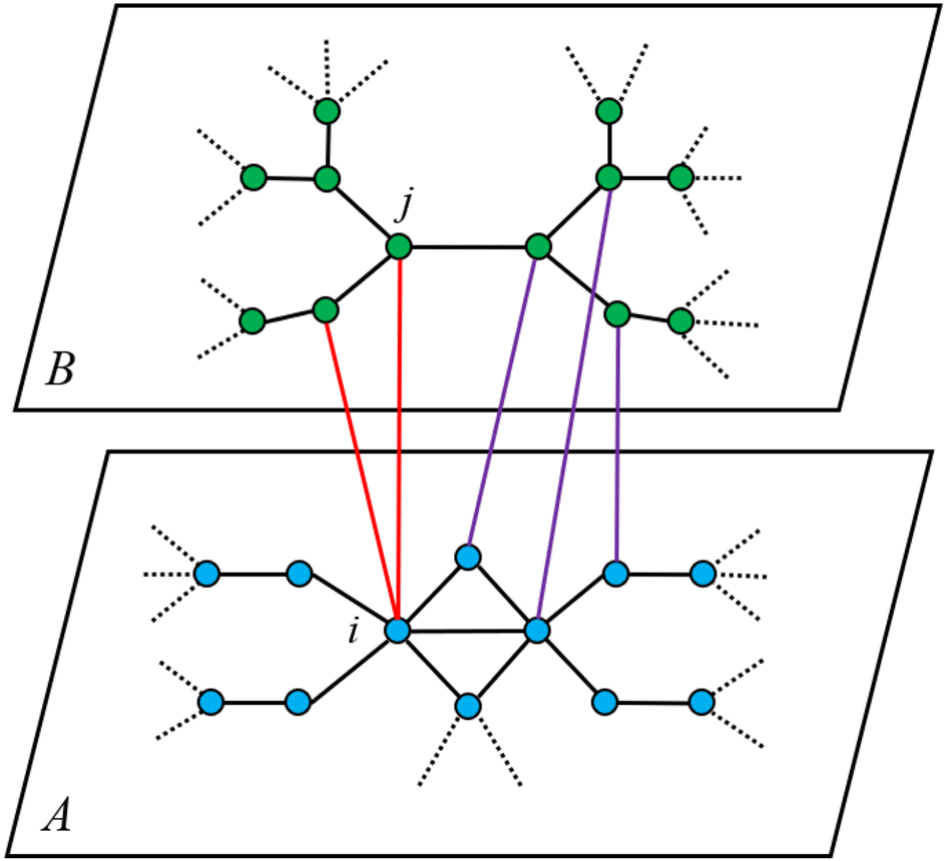
The diagram of interlayer relationship mapping in interdependent networks.

Mapping the node influence of the communication network to the power grid is a critical link. Under the premise of known dependencies, the ratio of the number of edges that a single communication node interacts with the power side accounts for the total number of dependent edges as the degree of influence of the communication node on the power node. Figure 7 shows the mapping relationship between dependent network layers. A and B represent the communication network and the power grid respectively. The red line represents the interaction edge between node *i* in the A network and the node in the B network. The number of red edges accounts for the total number of interactive edges, which is the degree of influence of node *i* on network B.

20 $$\begin{aligned} {F_{A - B}} = \frac{{\sum \nolimits _i^P {{w_{ij}}} }}{{\sum \nolimits _j^Q {\sum \nolimits _i^P {{w_{ij}}} } }}. \end{aligned}$$

Knowing the influence of the network nodes on both sides and the degree of influence between the two networks, this paper takes the control degree of the power node depending on the information communication node as the parameter of the mapping function and uses a linear mapping function to map the influence of the communication node to the connected nodes on the power node. Therefore, the influence of a power node is defined as

21 $$\begin{aligned} {\phi _A}({v_i}) = {\varphi _A}({v_i}) + {F_{A - B}} * {\varphi _B}({v_j}). \end{aligned}$$

### Evaluation indicator

After a cascading failure occurs, the robustness of the dependent network is measured by the ratio of the number of nodes in the largest connected graph to the total number of nodes, which is computed as follows:

22 $$\begin{aligned} p = \frac{{{{N'}_A} + {{N'}_B}}}{{{N_A} + {N_B}}}, \end{aligned}$$

where ${N_A}$ and ${N_B}$ represent the initial number of nodes in network A and network B, and ${N'}_A$ and ${N'}_B$ represent the number of nodes in the maximum connected graph in network A and network B after cascading faults, respectively.

According to the above formula, the ranking result $Rank\left[ {{v_i},\mathrm{{ }}MAFC\left( {{v_i}} \right) } \right]$ of the influential nodes is obtained. The node influence identification method is shown in Algorithm 1.
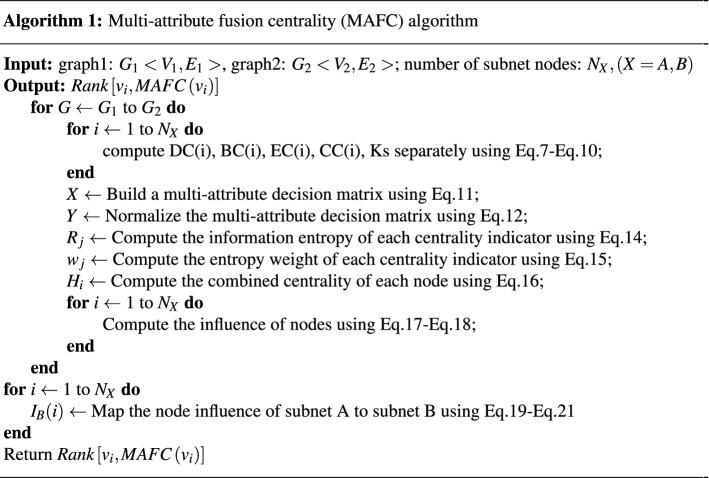


## Data Availability

The authors declare that all data supporting the findings of this study are included in this published article and its supplementary information files, or from the corresponding author upon request.

## References

[1] Song EY, FitzPatrick GJ, Lee KB, Griffor E (2022). A methodology for modeling interoperability of smart sensors in smart grids. IEEE Trans. Smart Grid.

[2] Shahid K (2021). On the use of common information model for smart grid applications—A conceptual approach. IEEE Trans. Smart Grid.

[3] Sun C-C, Hahn A, Liu C-C (2018). Cyber security of a power grid: state-of-the-art. Int. J. Electr. Power Energy Syst..

[4] Wu S (2022). Machine learning aided construction of the quorum sensing communication network for human gut microbiota. Nat. Commun..

[5] Li W (2021). Integrated inter-tower wireless communications network for terrestrial broadcasting and multicasting systems. IEEE Trans. Broadcast..

[6] Sadeghzadeh AM, Shiravi S, Jalili R (2021). Adversarial network traffic: Towards evaluating the robustness of deep-learning-based network traffic classification. IEEE Trans. Netw. Serv. Manag..

[7] Li X, Sun J-Q (2019). Multi-objective optimal predictive control of signals in urban traffic network. J. Intell. Transp. Syst..

[8] Gao J, Buldyrev SV, Stanley HE, Havlin S (2012). Networks formed from interdependent networks. Nat. Phys..

[9] Gao J, Buldyrev SV, Havlin S, Stanley HE (2011). Robustness of a network of networks. Phys. Rev. Lett..

[10] Liu X, Stanley HE, Gao J (2016). Breakdown of interdependent directed networks. Proc. Natl. Acad. Sci..

[11] Gao J (2022). Introduction to Networks of Networks.

[12] Buldyrev SV, Parshani R, Paul G, Stanley HE, Havlin S (2010). Catastrophic cascade of failures in interdependent networks. Nature.

[13] Advisers, E. Economic benefits of increasing electric grid resilience to weather outages. US Dept. Energy. Washington, DC, USA. Tech. Rep. (2013).

[14] Parshani R, Buldyrev SV, Havlin S (2010). Interdependent networks: Reducing the coupling strength leads to a change from a first to second order percolation transition. Phys. Rev. Lett..

[15] Huang X, Gao J, Buldyrev SV, Havlin S, Stanley HE (2011). Robustness of interdependent networks under targeted attack. Phys. Rev. E.

[16] Dong G, Gao J, Tian L, Du R, He Y (2012). Percolation of partially interdependent networks under targeted attack. Phys. Rev. E.

[17] Dong G (2013). Robustness of network of networks under targeted attack. Phys. Rev. E.

[18] Huang X (2013). The robustness of interdependent clustered networks. EPL (Europhys. Lett.).

[19] Wang S, Stanley HE, Gao Y (2018). A methodological framework for vulnerability analysis of interdependent infrastructure systems under deliberate attacks. Chaos Solitons Fractals.

[20] Motter AE, Lai Y-C (2002). Cascade-based attacks on complex networks. Phys. Rev. E.

[21] Gao Y-L, Chen S-M, Nie S, Ma F, Guan J-J (2018). Robustness analysis of interdependent networks under multiple-attacking strategies. Physica A.

[22] Wang N, Jin Z-Y, Zhao J (2021). Cascading failures of overload behaviors on interdependent networks. Physica A.

[23] Qi X, Yang G, Liu L (2020). Robustness analysis of the networks in cascading failures with controllable parameters. Physica A.

[24] Chen Z, Wu J, Xia Y, Zhang X (2017). Robustness of interdependent power grids and communication networks: A complex network perspective. IEEE Trans. Circuits Syst. II Express Briefs.

[25] Wang J, Jiang C, Qian J (2014). Robustness of interdependent networks with different link patterns against cascading failures. Physica A.

[26] Kim D-H, Motter AE (2008). Resource allocation pattern in infrastructure networks. J. Phys. A Math. Theor..

[27] Dou B-L, Wang X-G, Zhang S-Y (2010). Robustness of networks against cascading failures. Physica A.

[28] Chen C-Y, Zhao Y, Gao J, Stanley HE (2020). Nonlinear model of cascade failure in weighted complex networks considering overloaded edges. Sci. Rep..

[29] Bonacich P (1972). Factoring and weighting approaches to status scores and clique identification. J. Math. Sociol..

[30] Freeman LC (1977). A set of measures of centrality based on betweenness. Sociometry.

[31] Freeman LC (1978). Centrality in social networks conceptual clarification. Soc. Netw..

[32] Katz L (1953). A new status index derived from sociometric analysis. Psychometrika.

[33] Zhang Y, Shao C, He S, Gao J (2020). Resilience centrality in complex networks. Phys. Rev. E.

[34] Meghanathan N (2021). Neighborhood-based bridge node centrality tuple for complex network analysis. Appl. Netw. Sci..

[35] Rajeh C, Savonnet JC, Leclercq M, Cherifi M (2022). Comparative evaluation of community-aware centrality measures. Qual. Quant..

[36] Blöcker C, Nieves JC, Rosvall M (2022). Map equation centrality: Community-aware centrality based on the map equation. Appl. Netw. Sci..

[37] Li Z, Huang X (2022). Identifying influential spreaders by gravity model considering multi-characteristics of nodes. Sci. Rep..

[38] Wang B, Zhang J, Dai J, Sheng J (2022). Influential nodes identification using network local structural properties. Sci. Rep..

[39] Li Z, Huang X (2021). Identifying influential spreaders in complex networks by an improved gravity model. Sci. Rep..

[40] Shooshtarian L, Safaei F (2019). A maximally robustness embedding algorithm in virtual data centers with multi-attribute node ranking based on topsis. J. Supercomput..

[41] Hu J, Du Y, Mo H, Wei D, Deng Y (2016). A modified weighted topsis to identify influential nodes in complex networks. Physica A.

[42] Fei L, Deng Y (2017). A new method to identify influential nodes based on relative entropy. Chaos Solitons Fractals.

[43] Bian T, Hu J, Deng Y (2017). Identifying influential nodes in complex networks based on AHP. Physica A.

[44] Mo H, Deng Y (2019). Identifying node importance based on evidence theory in complex networks. Physica A.

[45] Chen C-Y, Zhao Y, Qin H, Meng X, Gao J (2022). Robustness of interdependent scale-free networks based on link addition strategies. Physica A.

[46] Congdong L, Yuan D, Zhifeng Y (2016). Dynamic information-based load reallocation strategy for cascading failure networks. J. South China Univ. Technol. (Nat. Sci.).

[47] Kitsak M (2010). Identification of influential spreaders in complex networks. Nat. Phys..

[48] Omar YM, Plapper P (2020). A survey of information entropy metrics for complex networks. Entropy.

[49] Chen D, Lü L, Shang M-S, Zhang Y-C, Zhou T (2012). Identifying influential nodes in complex networks. Physica A.

